# Do Water, Sanitation and Hygiene Conditions in Primary Schools Consistently Support Schoolgirls’ Menstrual Needs? A Longitudinal Study in Rural Western Kenya

**DOI:** 10.3390/ijerph15081682

**Published:** 2018-08-07

**Authors:** Kelly T. Alexander, Garazi Zulaika, Elizabeth Nyothach, Clifford Oduor, Linda Mason, David Obor, Alie Eleveld, Kayla F. Laserson, Penelope A. Phillips-Howard

**Affiliations:** 1 Liverpool School of Tropical Medicine, Liverpool L35QA, UK; Garazi.Zulaika@lstmed.ac.uk (G.Z.); Linda.Mason@lstmed.ac.uk (L.M.); Penelope.Phillips-Howard@lstmed.ac.uk (P.A.P.-H.); 2 Cooperative for Assistance and Relief Everywhere (CARE), 151 Ellis St NE, Atlanta, GA 30303, USA; 3 Kenya Medical Research Institute, Kisumu 1578-40100, Kenya; ENyothach@kemricdc.org (E.N.); oduor.clifford@gmail.com (C.O.); DObor@kemricdc.org (D.O.); kel4@cdc.gov (K.F.L.); 4 Safe Water and AIDS Programme, Kisumu 3323-40100, Kenya; alie@swapkenya.org; 5 Centers for Disease Control and Prevention, Atlanta, GA 30333, USA

**Keywords:** water, sanitation, hygiene, menstruation, MHM (menstrual hygiene management), schools, rural, Kenya, girls

## Abstract

Many females lack access to water, privacy and basic sanitation—felt acutely when menstruating. Water, sanitation and hygiene (WASH) conditions in schools, such as access to latrines, water, and soap, are essential for the comfort, equity, and dignity of menstruating girls. Our study was nested within a cluster randomized controlled pilot feasibility study where nurses provided menstrual items to schoolgirls. We observed the WASH conditions of 30 schools from June 2012–October 2013 to see if there were any changes in conditions, to compare differences between study arms and to examine agreement between observed and teacher-reported conditions. Data came from study staff observed, and school teacher reported, WASH conditions. We developed scores for the condition of school facilities to report any changes in conditions and compare outcomes across study arms. Results demonstrated that soap availability for students increased significantly between baseline and follow-up while there was a significant decrease in the number of “acceptable” latrines. During the study follow-up period, individual WASH indicators supporting menstruating girls, such as locks on latrine doors or water availability in latrines did not significantly improve. Advances in WASH conditions for all students, and menstrual hygiene facilities for schoolgirls, needs further support, a defined budget, and regular monitoring of WASH facilities to maintain standards.

## 1. Introduction

Menstrual hygiene has been recognized as essential for girls’ dignity and basic rights, and key to ensuring equity in health and educational outcomes [1]. Menstrual hygiene challenges for women and girls must be addressed in order to achieve Sustainable Development Goals 5 (gender equality) and 6 (water and sanitation for all).

The WHO Joint Monitoring Programme definition of menstrual hygiene management (MHM) points to the need for women and girls to have access to water and soap for body and hand washing, sanitation options that are clean and private, with disposal amenities, and access to clean menstrual hygiene materials, both at home and away from home [2]. Approximately 1.5 billion females (aged 15–49) are menstruating each month [3] with 300 million menstruating each day [4]. Some evidence suggests that indicators of open defecation (not using a toilet or latrine) and limited access to water and soap at home (lack of water on the premises, and lack of a handwashing facility with soap), may represent the global burden of females who are unable to access privacy and sufficient water and soap during their menstruation [5]. A study from Nigeria, suggests this is not fully accurate—as women without sanitation facilities at their home may still have access to a private place to change their absorbent materials [6]. Although there is wide variation in facility access between and within countries, it is estimated that at least half a billion women and girls are unable to manage their menstruation in privacy due to the lack of access to a toilet or latrine [5]. The numbers may be lower [6], or higher, as these indicators do not take into account the cultural taboos and practices around menstruation that may limit the mobility and/or safety of females when menstruating [7,8].

In low and middle income countries, a number of studies have found links between poor menstrual knowledge, limited access to absorbent materials and inadequate facilities at school with girls’ absenteeism [9,10,11,12]. There are also a number of quantitative studies that found no association between improved MHM and reduced absenteeism [13,14,15]. These findings may be due to poor quality attendance records, irregular menstruation among adolescent girls, girls missing school for hours (not days captured on attendance records), and the high potential for girls not to report menstrual-related issues as reasons for absence [16]. Discussions with teachers, parents, girls and boy students report the negative effects of menarche for girls. Examples described include absenteeism and reduced performance at school, and not performing regular activities, such as collecting water or participating in religious rituals [17,18,19,20,21,22,23,24,25]. Other consequences of poor menstrual management options include not participating in class or school sports, engaging in transactional sex for access to pads, feeling worried or depressed, low self-esteem and pregnancy [18,20,21,26,27,28,29,30]. Potential effects of menstrual practices on the reproductive tract, including sexually transmitted infections, have been reported in an impoverished setting in Kenya [11].

Adequate menstrual management options for girls in schools means they have access to services, facilities and products to manage their menstruation with dignity, safety, and privacy. Educating other females (e.g., mothers and teachers), men (e.g., fathers and teachers), and boys (e.g., brothers and peers) on menstruation, and addressing cultural taboos, can also benefit menstruating girls in feeling comfortable and gaining more access to the items they need [25,28,31,32].

The physical aspects of the water, sanitation and hygiene (WASH) environment and facilities in schools available to girls is one essential component of MHM [22,33,34,35,36,37]. However, few WASH studies have longitudinally followed schools to examine trends in facility conditions and hygiene supplies over time, with specific attention to what is available for menstruating girls. This report presents WASH-related data collected over a 15-month period. The larger study involved 30 schools participating in a cluster randomized controlled pilot feasibility study which provided menstrual products to schoolgirls [11,18,38]. The study arms consisted of three sets of ten schools randomly allocated for eligible girls to receive a menstrual cup, sanitary pads or no menstrual item (control). The objectives of this study were to see if there was any change in the schools’ WASH conditions between baseline and average follow-up and across all 5 follow-up rounds, as well as to see if there was a difference in WASH conditions between intervention arms. A difference in conditions was anticipated in cup, and potentially pad schools since the importance of hand and menstrual hygiene was stressed with girls, parents and teachers in these schools. The study also examined if school informants’ reporting of WASH and MHM facilities and conditions accurately portrayed the situation in the schools surveyed, by comparing with observations made by study staff. This paper reports WASH conditions observed by study staff and described by school informants over five follow-up visits.

## 2. Materials and Methods

### 2.1. Study Site and Population

The study was conducted in Gem, Siaya County, formerly part of Nyanza Province in rural western Kenya. The study site was within a health and demographic surveillance system providing socio-demographic and epidemiological data on the ~70,000 persons living in Gem [39]. The population are ethnically Nilotic Luo, predominantly farmers and fishers who are polygynous and living in compounds comprising a central house with smaller houses for wives and children [40]. The main water sources for Nyanza Province are borehole, spring or well (45.9%) and stream (29.9%) [41] and over 75% of houses reportedly have basic latrines [42]. The most common diseases are malaria (54% of illnesses), respiratory infections (15%) and diarrheal disease (4%), with 22% of children stunted in Siaya County [42].

### 2.2. Study Design and School Sampling

This WASH study was nested within a larger cluster randomized controlled pilot feasibility study [11]. This study was designed as a repeat cross-sectional WASH survey across eligible study schools. The sample was derived from the District Education Department school list for 2012. Of 71 schools in the study area, representatives from 62 attended an information meeting, agreed to participate in a WASH survey, and met our eligibility criteria (all girl or co-education day schools with classes 5 through 8). The remaining nine schools that did not come to the initial meeting were not contacted further. In June 2012, our staff conducted a WASH survey in each of the 62 schools during an unannounced visit [38]. Forty-one schools met our WASH conditions inclusion criteria: separate latrine bank for girls, at most 70:1 students for each latrine, and water for handwashing observed to be present at school on the day of the unannounced survey visit, and 30 schools were randomly selected for the study.

Separate latrines for girls, “lower” student to latrine ratios, and school access to water are three indicators considered to demonstrate improved WASH conditions for less economically developed settings [13,43,44]. For the larger menstrual solutions study we looked for schools with baseline improved WASH conditions in order to maintain focus on the feasibility of menstrual solutions for schoolgirls. Schools were followed-up at 5 distinct time points between November 2012 and October 2013, at 2 to 3 month intervals.

### 2.3. Menstrual Study Interventions

The thirty schools were block randomized pre-intervention into three equal arms of ten schools: (1) menstrual cup (Mooncups^®^, an insertable, reusable bell-shaped container); (2) sanitary pads (Always^®^, two packs monthly); or (3) no menstrual management item. Schoolgirls were eligible to participate in the trial if they were 14–16 years old, had no precluding disability, had experienced at least three menses, were resident for at least 4 months in the study area, and provided written assent (schoolgirl) and consent (parents/caregivers) [11]. Henceforth schools are referred to as cup, pad, or usual practice. Study nurses were assigned to three schools each to distribute the menstrual intervention, (where applicable), survey and counsel girls, and to provide powdered detergent to all schools each term with instructions for making soapy water available for handwashing school-wide. A short instruction manual on basic WASH facility maintenance was also provided to all schools after baseline observations. The manual covered topics ranging from doors and locks on latrines, creating schedules for latrine cleaning and providing water for handwashing through the entire school day.

### 2.4. Data Collection

Between June 2012 and October 2013 enrolled schools received one WASH baseline and five WASH follow-up visits. All visits were unannounced to ensure data collection reflected typical conditions. During these visits trained WASH study staff conducted a semi-structured interview with the head teacher (or designated representative) on school WASH conditions, and then independently observed and recorded current WASH conditions in the school. All data were collected electronically using netbooks (2goTM Convertible Classmate PC). The number of students enrolled in each class was recorded at baseline in order to calculate student to latrine ratios.

Data included information on the school’s primary water source, as well as the availability of water, soap, cleaning supplies and sanitary pads at the school. Separate independent WASH observations by study staff included the physical conditions of latrines, presence of washing water inside the girls’ latrines, and presence of water and soap at the school (see: Supplementary Material File S1 for Electronic School WASH Survey). For latrines, staff recorded the stability of the floor or platform, and the presence of: (1) holes in the wall; (2) an offensive smell; (3) feces or urine pooled on the floor; (4) a door; and (5) a working inside lock. The presence of handwashing water and soap for students was also observed and recorded upon arrival at the school. Defining and agreeing on “offensive smell” and “clean” latrines were integral parts of a three-day WASH training to reduce observer bias of field staff.

### 2.5. Study WASH/MHM Indicators and Definitions

The number of latrines used for data analysis included only those exclusively for girls or boys, excluding latrines intended for teachers or unidentified. A target pupil-latrine ratio was set at 25:1 for girls and 30:1 for boys, following the Kenyan government guidelines [45]. Beyond student to facility ratios, we defined “acceptable” latrines for girls as those with the following observed conditions: clean (no visible feces on floor), no strong/offensive smell, containing door and roof, no major holes in walls, stable floor or stable latrine slab. Locks were not included in the criterion for acceptable latrines due to the limited number of locks observed. Hygienic latrines were those judged as clean because they had no visible feces or urine on the floor. Schools having a private place for girls to wash or change for MHM was defined by having a washing or changing room designated for girls, which at minimum comprised of one girls’ stall (toilet or changing room) with a lockable door. Presence of water was considered essential for girls’ MHM.

### 2.6. Data Processing and Analysis

In order to compare schools, WASH and MHM scores were calculated for each school based on data observed. WASH scores between 0–3 were created with equal weight given to the following three observed variables: (1) water observed available for handwashing; (2) soap observed available for handwashing; (3) rank of girl to acceptable latrine ratio. Girl to acceptable latrine ratios were ranked and assigned a score according to distribution quartile: schools with 0–30 girls per acceptable latrine received 1 point; 30.1–45 girls per latrine were assigned 0.75 points; 45.1–75 received 0.5 points, and schools with 75.1–140 girls per acceptable latrine received 0.25 points. Schools with ratios over 140 girls per acceptable latrine and those schools that had no acceptable latrines received 0 points.

The calculated MHM score ranged from 0–3 with equal weight given to the following three observed variables: (1) presence of washing water at girls’ latrine; (2) privacy wall at girls’ latrine, and (3) at least one private (lockable) place for girls within the school (that girls could use to change pads or clothes). We also calculated a WASH + MHM score that combined the six variables from the WASH and MHM scores with a possible max score of 6.

Cochrane Mantel-Haenszel (CMH) tests were employed to provide prevalence estimates on school level variables and compare the three treatment groups for categorical variables, while for continuous variables Kruskal-Wallis one-way analysis of variance tests were used to determine if any differences existed between the three treatment groups. We averaged each school’s WASH and latrine indicators across follow-up rounds due to the small sample sizes within each individual treatment group (*N* = 10). For WASH, MHM, and WASH + MHM scores a count generalized linear mixed effects model with an autoregressive error was employed to assess if there was improvement or deterioration of conditions over the 5 follow-up rounds [46,47,48]. Wald F tests were run on the aggregated scores as well as their individual indicators. Paired *t*-tests were used to analyze whether differences were seen between baseline and mean follow-up for school level variables. Lastly, Kappa coefficients were used to look at agreement between reported and observed WASH measures. Probabilities of *p* < 0.05 were considered statistically significant. Analysis was conducted in SAS 9.3 (SAS Institute, Cary, NC, USA) and STATA 13 (Stata Corp., College Station, TX, USA).

### 2.7. Ethical Considerations

The Kenyan Medical Research Institute, (SSC No 2198), LSTM, (12.11) approved this research, with exemption reliance on KEMRI ethical board from the U.S. CDC Institutional Review Board (Centers for Disease Control and Prevention). Head teachers gave oral consent for both the semi-structured interview and school observations at the beginning of each visit.

## 3. Results

### 3.1. Baseline

At baseline there were a total of 409 student sanitation facilities (374 latrines, 35 urinals) and 12,947 students (6248 girls, 6699 boys) across the 30 study schools. The average student to latrine ratio rates for girls was 37:1 (Table 1), with only one school designating a urinal for girls. At baseline, 63% of schools had a greater number of girls per latrine than was allowed by the Government of Kenya (GoK) standard of 25 girls or less per latrine. The average boys to latrine/urinal ratio was 38:1, with over half (53%) of schools adhering to the GoK standard for boys of 30:1. Wide variability was seen across individual schools in the number of WASH facilities available to students, however there were no significant differences in baseline school characteristics between study arms (Table 1).

### 3.2. Acceptable and Hygienic Latrines at Baseline and Follow-up and Across Arms

For the remainder of the paper, results and discussion of latrines at the school refer to girl-latrines only. Restricting latrines to those designated as acceptable reduced the number of schools that contributed data to the analysis in each follow-up round as some schools had no latrines in acceptable conditions, thus the worst-performing schools are not represented. When looking at only acceptable latrines, the girl to acceptable latrine ratio jumped to an average of 64:1 at baseline, across all schools (i.e., 64 girls to 1 latrine in acceptable condition; Table 2) as compared to 37 girls per latrine when accounting for latrines in any condition (Table 1). This acceptable latrine ratio decreased to 56 girls per acceptable latrine across all schools during follow-up when the number of acceptable latrines was averaged across the five rounds of data collection. At baseline in cup schools the ratio was significantly better compared to pad and usual practice schools at 29:1 girls per acceptable latrine compared to 79 and 70 girls, respectively (*p* = 0.0278). During follow-up, cup schools saw the ratio increase, rising to 43:1 girls per acceptable latrine across all rounds, while ratios fell for pad (56) and usual practice schools (69). Overall, there was a significant deterioration of latrine conditions from baseline to follow-up. At baseline, 83% of all schools had at least one latrine that met all criteria to be classified as acceptable, while only 53% of all schools did during follow-up rounds (*p* = 0.0046). At baseline 53% of study schools had at least one lockable latrine for girls to use, while during follow-up only 23.3% of study schools had at least one lockable latrine (*p* = 0.0010). For hygienic latrines, having no visible feces or pooled urine, 100% of schools had at least one hygienic latrine at baseline, whereas on average during follow-up 73.3% of school visits had at least one hygienic latrine (*p* = 0.0029). Usual practice schools saw the greatest decrease from baseline with an average of 60% of school visits during rounds 1–5 having at least one hygienic latrine for girls (*p* = 0.0368), as seen in Table 2.

### 3.3. School WASH Conditions at Baseline and Follow-Up and across Arms

During the study period, school handwashing and cleaning supplies overall did not significantly vary across arms or change between baseline and average follow-up. In follow-up rounds, availability of soap for handwashing was more frequently reported by teachers as well as observed by study staff, increasing from 0% of observed schools supplying soap at baseline to over 26% of schools on average during follow-up supplying soap for handwashing on the day of the unannounced visit (Table 3). This improvement in soap provision was significant for all study arms between baseline and average follow-up, and for cups and usual practice schools when looking at improvements over the five follow-up rounds only. The reported presence of cleaning supplies for latrines improved throughout the course of the study, although this was only significant in the pad group both across the five follow-up rounds and also when compared against their baseline conditions. Overall, privacy walls in girl’s latrines increased between baseline and average follow-up for cup and pad schools, while usual practice schools saw a drop in privacy walls throughout the course of the study. Less than half of all schools during follow-up had a private place for girls to change or wash, and less than one third of schools had washing water observed at girls’ latrines. While these conditions improved when compared to baseline for pad and usual practice schools, cup schools saw a significant decline in private changing places throughout the five follow-up rounds.

### 3.4. School WASH and MHM Scores

Schools appeared to improve slightly in WASH and MHM scores overall across the study period. For WASH, schools scored higher at average follow-up when compared to baseline; however, these improvements were not statistically significant. No school received all 3 points comprising the WASH score, with schools on average receiving 1.5 points at baseline (Table 4). In comparison, schools obtained lower scores on the MHM assessment, on average scoring 1.1 out of 3 points. Cup schools showed the most improvements in the MHM score at average follow-up, but this was not significant compared to baseline conditions (Table 4). Significant improvements were not seen between baseline and average follow-up in the WASH or MHM scores individually, but when combined an overall improvement was seen across all schools (*t* = 2.28, *p* = 0.030) (Table 4). When looking at change during follow-up longitudinally, WASH scores saw significant improvement across all study schools (*p* = 0.014).

### 3.5. Observed and Reported Conditions

For WASH and MHM scores in the above section, score calculations were based on observations by study staff. In addition, data from school staff on select WASH and MHM indicators was also collected and compared to observed data. There was significant variability in responses between study staff observation and school staff report (Table 5). Teachers in all study schools reported higher levels of available handwashing water, soap, and within latrine washing water compared to what was physically observed by study staff. Overall across all schools the concordance between reported water for handwashing and observed water showed no agreement (*k* = −0.130, *p* < 0.001). For soap there was a significant difference in all study arms between what was seen and what was reported, with school staff significantly more likely to report the availability of soap compared with that observed by study staff (cup *p* < 0.001; pad *p* < 0.012; usual practice *p* = 0.016). For the presence of washing water in girls’ latrines there was a difference between what was observed and what was reported, however this difference was only significant in usual practice schools (*p* = 0.035). Significant disagreement was seen across all three indicators (handwashing water, soap for handwashing, washing water in latrines) when looking at all schools together comparing observed conditions and reported conditions. However, for 87% of the usual practice schools, there was no significant disagreement between reported and observed handwashing water.

## 4. Discussion

This is the first study to present quantitative data on WASH conditions in schools within the context of a menstrual product intervention study. The objectives of this study were to evaluate WASH facility conditions over time and to see if there was any difference in WASH conditions between study arms. We additionally looked at differences in observed and reported WASH conditions. We used the concept of an “acceptable” latrine to discuss the sanitation facilities that provided girls with a safe, clean and private place to change. On average, all schools improved slightly from baseline conditions in terms of their combined WASH + MHM score. While improvements appeared slightly better in the cup and pad schools, there was no significant difference. These minor improvements may be due to the presence of study staff and nurses, increasing the emphasis school leadership placed on WASH and MHM facilities for girls. It is important to note that study-eligible schools included those with less than 70 students per latrine, handwashing water provided at the first unannounced visit, and separate latrines for girls, all indicators of improved WASH conditions, particularly for girls [12,43,44,49]. Although these 30 schools had better WASH conditions at baseline than other government primary schools in the area, conditions were not ideal to support basic WASH due to inconsistent provision of water, soap and clean latrines.

Observed soap available for student handwashing significantly improved across all schools—which was expected since there was provision of powdered detergent on a monthly basis to all schools (quantity of soap depended on size of the school population). This significant increase is misleading however, since 0% of schools provided soap at baseline and soap was observed only about 30% of the time across the five follow-up rounds. Although soap availability improved significantly, it did not improve consistently across all schools. On average during follow-up 80–90% of schools were observed with handwashing water for students, falling from 100% handwashing water available at baseline. Rainfall (representing water availability) in the area was approximately the same for June 2012 and October 2013 [50].

For boys and girls, latrine ratios were, on average, within the Kenyan government standard of 30:1 and 25:1 respectively. However, when looking only at acceptable latrine conditions for girls, the ratio was often more than double the number of schoolgirls per latrine within the GoK standard (except for cup schools at baseline: 29 girls per acceptable latrine, for which we have no explanation since allocation to arms was random). A paper by Garn et al. [51] found that students are more likely to use latrines when student to latrine ratios were lower, and that girls (not boys) were probably more likely to use latrines that were clean and in good condition—validating the idea of “acceptable” latrines for girls as key. Locks were rarely seen inside girls’ latrines, and therefore were not included in the definition of an acceptable latrine, despite evidence that locks are essential for the privacy and comfort of girls when using the latrine or when changing at school [22,37].

Cup arm girls at baseline had access to at least one acceptable latrine. During follow-up rounds, only 60% of school visits had at least one acceptable latrine—meaning that 40% of the time cup arm girls had no acceptable latrines. Despite limited access to soap and non-ideal, non-hygienic latrine conditions, there were no adverse outcomes for any girls in any of the study arms [13]. Insertable menstrual cups, just like disposable sanitary pads or cloth/rags (“usual practice”) can be used in schools and homes where soap and hygiene practices are limited (home WASH survey data from this study, unpublished). A study in Nigeria found that women often do not manage their menstruation in improved sanitation facilities, which lines up with our finding that soap, water and clean sanitation facilities (specifically at home) are not necessarily needed for comfortable menstrual management [6].

Conditions in schools somewhat improved over the course of the study, but were still not ideal for basic WASH access and practice [52]. Schools did not consistently have facilities that would allow girls to easily manage their menstruation, including provision of water and soap. These findings suggest WASH conditions remain inadequate for students and menstruating girls in particular, even within the context of a closely monitored study, with study nurse presence, study provision of handwashing soap to schools, and school WASH guidelines given to all head teachers post-baseline. There was a significant discrepancy between the conditions observed and the conditions reported. Beyond interpreting this as courtesy bias, the differences between reported and observed WASH and MHM conditions could indicate that teachers see handwashing water, soap and washing water as key elements of services which should be available to students, but that external resources or support from the Ministry of Education and school stakeholders is needed.

Although not reported in this study, there is a concern about lack of disposal options for menstrual hygiene products in schools [53]. In many school settings girls do not have bins to dispose of used menstrual items [54]. Used pads or rags are generally put in latrine pits, increasing the frequency of need for pit emptying, and potentially disrupting the functionality of latrine exhausters [54,55]. The lack of disposal options for girls in rural schools may affect girls’ comfort and comes at a cost for the schools and for private sanitation providers. Future research and school WASH programming should consider disposal options at schools as part of the required infrastructure to support menstruating schoolgirls.

If teacher understanding and priority is already present, then it is likely that schools need a specific budget for repairs, upgrades and maintenance [52] along with motivation from the Ministry of Education for consistent monitoring [56] and higher prioritization for WASH and menstrual needs from the parent or school management association. Parents, teachers and students all need more education on puberty, reproductive health and menstruation, so that the needs of females can be better understood and met in the school (and home) environment.

## 5. Limitations

We “averaged” WASH and latrine conditions across schools in each arm and across rounds due to the small sample size within each intervention group, which may have limited the evidence that some individual schools made great strides to improve WASH conditions during the study. It is also important to note that since we only included schools with water available, separate girl latrines and 70 or less students per latrine, our findings are not broadly applicable. Additionally, the WASH and MHM scores were calculated from three variables each—limiting the extent to which variation in scores across schools could be explained. Although outside the scope of this paper, it is important to note that we are only discussing the needs of girls in-school, recognizing there are essential menstrual hygiene needs away from school, and among school-age girls that are not attending school [57,58].

## 6. Conclusions

Water, sanitation and hygiene conditions in schools improved, on average, during the study follow-up period, but not strongly enough to impact menstruating girls’ access to sufficient WASH facilities at schools, with no significant difference between arms. Soap distribution to schools increased soap provision for students, but this was inconsistent. Monitoring actual school latrine conditions may be required, as opposed to only considering student to latrine ratios or teacher-reported WASH services. Improvements in the conditions of WASH for all students, and MHM facilities for girls in schools needs further support and prioritization. Only once the needs of menstruating girls in schools are recognized and addressed will we be on our way to providing water and sanitation for all and achieving gender equality.

## Figures and Tables

**Table 1:** Baseline characteristics of 30 schools in western Kenya June 2012.

School Characteristics	Cup	Pad	U.P.	All Schools	Differ Across Groups
	(*n* = 10; *N* = 10)	(*n* = 10; *N* = 10)	(*n* = 10; *N* = 10)	(*n* = 30; *N* = 30)	Kruskal Wallis X^2^
	Mean, SD (range)	Mean, SD (range)	Mean, SD (range)	Mean, SD (range)	*p*-Value
No. full-time teachers	8.5, 0.9 (7–10)	10.7, 2.5 (8–14)	10.9, 4.2 (6–21)	10.03, 3.0 (6–21)	0.1224
No. part-time teachers	3.1, 1.0 (2–5)	2.6, 0.7 (2–4)	3.0, 0.8 (2–4)	2.91, 0.9 (2–5)	0.5287
No. in-use classrooms	8.4, 1.1 (6–10)	9.1, 1.7 (8–12)	10.8, 3.6 (8–19)	9.43, 2.5 (6–19)	0.1423
No. girls	175.1, 73.5 (90–283)	219.3, 98.1 (119–403)	230.4, 123.2 (78–519)	208.3, 99.8 (78–519)	0.5274
No. boys	187.9, 65.3 (100–302)	231.0, 102.6 (113–406)	251.0, 140.0 (76–588)	223.3, 106.7 (76–588)	0.4393
No. student latrines	11.4, 5.2 (4–19)	13.3, 4.2 (8–20)	12.7, 5.1 (8–24)	12.5, 4.7 (4–24)	0.7388
No. student urinals	1.1, 1.0 (0–3)	1.2, 1.1 (0–4)	1.2, 1.2 (0–4)	1.17, 1.1 (0–4)	0.9967
Girls: latrine ratio	36.8, 18.0 (17–67)	37.0, 18.0 (13–68)	37.8, 19.9 (11–70)	37.2, 18.0 (11–70)	0.9961
Boys: latrine + urinal ratio	31.6, 10.2 (16–50)	38.78, 30.4 (9–102)	42.14, 25.5 (19–98)	37.50, 23.2 (9–102)	0.7617
Total student:latrine ratio	34.7, 10.0 (24–53)	36.7, 19.9 (12–74)	39.4, 18.0 (15–75)	36.96, 16.1 (12–75)	0.7742
Total student: latrine + urinal	32.1, 10.8 (23–53)	33.8, 18.3 (11–67)	36.1, 15.6 (14–67)	34.0, 14.8 (11–67)	0.7783

*n* = number of visits to schools; *N* = Number of schools; U.P. = usual practice.

**Table 2:** Girls’ latrines across arms, baseline and average over five follow-up rounds in schools in western Kenya 2012–2013.

Latrine Indicators	Baseline Conditions (Rd 0)					Average Conditions (Rd 1–5)					Change from Baseline to Average Follow-up			
	Cup	Pad	U.P.	All Schools	Differ Accross Groups	Cup	Pad	U.P.	All Schools	Differ Accross Groups	Cup	Pad	U.P.	All Schools
	*n* = 10 *N* = 10	*n* = 10 *N* = 10	*n* = 10 *N* = 10	*n* = 30 *N* = 30		*n* = 50 *N* = 10	*n* = 50 *N* = 10	*n* = 50 *N* = 10	*n* = 150 *N* = 30		*n*= 60 *N* = 10	*n*= 60 *N* = 10	*n*= 60 *N* = 10	*n*= 180 *N* = 30
	Mean, SD (range)	Mean, SD (range)	Mean, SD (range)	Mean, SD (range)	Kruskal Wallis X^2^ *p*-value	No. (%)	No. (%)	No. (%)	No. (%)	Kruskal Wallis X^2^ *p*-value	Paired *t*-test, *p*-value	Paired *t*-test, *p*-value	Paired *t*-test, *p*-value	Paired *t*-test, *p*-value
No. acceptable girls latrines	3.5, 3.7 (0–11)	3.6, 2.9 (0–8)	3.9, 2.3 (1–7)	3.7, 2.9 (0–11)	0.8768	4.2, 2.5 (0–10)	3.9, 2.7 (0–13)	3.5, 2.9 (0–10)	3.9. 2.7 (0–13)	0.3486	0.519	0.7162	0.5418	0.6795
Girl: acceptable latrine ratio among schools with at least 1 acceptable latrine	28.9, 6.8 (21.6–36.8)	79.5, 52.2 (20.8–170)	70.4, 37.3 (26–140)	63.7, 43.0 (20.8–170)	0.0278 *	43.2, 27.2 (20.2–141.5)	56.1, 35.6 (14.9–137)	69.1, 55.5 (13–280)	55.6, 41.6 (13–280)	0.1003	0.1422	0.262	0.899	0.5243
	**Count (%)**	**Count (%)**	**Count (%)**	**Count (%)**	**Kruskal Wallis X ^2^** ***p*-value**	**Count (%)**	**Count (%)**	**Count (%)**	**Count (%)**	**Kruskal Wallis X ^2^** ***p*-value**	**Paired *t*-test,** ***p*-value**	**Paired *t*-test,** ***p*-value**	**Paired *t*-test,** ***p*-value**	**Paired *t*-test,** ***p*-value**
No. schools with at least one acceptable latrine (%)	6 (60%)	9 (90%)	10 (100%)	25 (83.30%)	0.0490 *	6 (60%)	8 (80%)	2 (20%)	16 (53.3%)	0.0266 *	1	0.3434	0.0002 *	0.0046 *
No. schools with at least one lockable latrine (%)	6 (60%)	4 (40%)	6 (60%)	16 (53%)	0.5958	1 (10%)	3 (30%)	3 (30%)	7 (23.3%)	0.4865	0.0522	0.5911	0.0811	0.0100 *
No. schools with at least one hygienic latrine ^††^ (%)	10 (100%)	10 (100%)	10 (100%)	30 (100%)	1	7 (70%)	9 (90%)	6 (60%)	22 (73.3%)	0.3156	0.0811	0.3434	0.0368 *	0.0029 *

^††^ Hygienic latrine were those observed to be clean (no visible feces or pooled urine), * Significant difference; *n* = number of visits to schools; *N* = Number of schools; U.P. = usual practice.

**Table 3:** School water, sanitation, and hygiene (WASH) conditions at baseline and average over five follow-up rounds, and comparisons western Kenya 2012–2013.

WASH Indicators	Baseline Conditions (Rd 0)				Average Conditions (Rd 1–5)				Change from Baseline to Average Follow-up			Change Across 5 Follow-up Rounds		
	Cup	Pad	U.P.	Differ Across Groups	Cup	Pad	U.P.	Differ Across Groups	Cup	Pad	U.P.	Cup	Pad	U.P.
	*n* = 10 *N* = 10	*n* = 10 *N* = 10	*n* = 10 *N* = 10		*n* = 50 *N* = 10	*n* = 50 *N* = 10	*n* = 50 *N* = 10		*n* = 60 *N* = 10	*n* = 60 *N* = 10	*n* = 60 *N* = 10	*n* = 50 *N* = 10	*n* = 50 *N* = 10	*n* = 50 *N* = 10
**Observed WASH**	**No.** **(%)**	**No.** **(%)**	**No.** **(%)**	**CMH** ***p*-value**	**No.** **(%)**	**No.** **(%)**	**No.** **(%)**	**CMH** ***p*-value**	**Paired *t*-test,** ***p*-value**	**paired *t*-test,** ***p*-value**	**paired *t*-test,** ***p*-value**	**Wald *F* test,** ***p*-value**	**Wald *F* test,** ***p*-value**	**Wald *F* test,** ***p*-value**
Water for handwashing (HW)	10 (100%)	10 (100%)	10 (100%)	†	42 (84%)	40 (80%)	45 (90%)	0.3794	0.0548	0.0319 *	0.3434	0.2653	0.2019	1.0
Soap for HW	0 (0%)	0 (0%)	0 (0%)	1.0000	13 (26%)	14 (28%)	15 (30%)	0.9062	0.0037 *	0.0165 *	0.0030 *	0.0100 *	0.0585	0.0453 *
Girls’ separate latrine bank	10 (100%)	10 (100%)	10 (100%)	†	47 (95.9%)	50 (100%)	50 (100%)	0.1281	0.1679	1.0000	1.0000	0.5807	1.0000	1.0000
Privacy wall at girls’ latrine	4 (40%)	6 (60%)	6 (60%)	0.5958	26 (53.1%)	36 (72%)	24 (48%)	0.0387 *	0.4417	0.4344	0.2393	0.8989	0.5951	0.3758
Washing water at girls’ latrine	3 (30%)	1 (10%)	2 (20%)	0.5465	12 (24.5%)	14 (28%)	13 (26%)	0.9241	0.7304	0.2247	0.3434	0.7148	0.4028	0.4152
Private place to change or wash	5 (50%)	3 (30%)	3 (30%)	0.5741	23 (46.9%)	24 (48%)	17 (34%)	0.2928	0.7263	0.1823	0.5911	0.0442 *	0.5193	0.4846
**Reported WASH**	**No.** **(%)**	**No.** **(%)**	**No.** **(%)**	**CMH** ***p*-value**	**No. (%)**	**No.** **(%)**	**No.** **(%)**	**CMH** ***p*-value**	**Paired *t*-test,** ***p*-value**	**Paired *t*-test,** ***p*-value**	**Paired *t*-test,** ***p*-value**	**Wald *F* test,** ***p*-value**	**Wald *F* test,** ***p*-value**	**Wald *F* test, *p*-value**
School “Always” supplies HW water	10 (100%)	5 (50%)	8 (80%)	0.3416	48 (96%)	46 (92%)	45 (90%)	0.1335	0.1679	0.3434	0.1679	0.0785	1.00	1.00
HW water today	9 (90%)	8 (80%)	8 (80%)	0.7929	45 (90%)	43 (86%)	46 (92%)	0.7468	1.0000	0.6637	0.3938	0.4648	0.3025	0.9081
School “Always” supplies soap	1 (10%)	1 (10%)	0 (0%)	0.5958	37 (74%)	30 (60%)	30 (60%)	0.2417	0.0017 *	0.0009 *	0.0011 *	<0.001 *	0.0014 *	0.0016 *
Soap for HW today	0 (0%)	1 (10%)	0 (0%)	0.3679	31 (62%)	25 (50%)	23 (46%)	0.4226	<0.001 *	0.0296 *	<0.001 *	<0.001 *	0.2424	0.0100
School “Always” has latrine cleaning supplies	1 (10%)	2 (20%)	4 (40%)	0.8704	20 (40%)	13 (26%)	19 (38%)	0.2706	0.0852	0.0343 *	0.0629	0.0858	0.0307 *	0.1869
Latrines cleaning supplies today	1 (10%)	2 (20%)	3 (30%)	0.5465	24 (48%)	27 (54%)	27 (54%)	0.7876	0.0550	0.710	0.2647	0.1233	0.9748	0.4604
Washing water in girls’ latrines	7 (70%)	5 (50%)	8 (80%)	0.3624	38 (76%)	37 (74%)	28 (56%)	0.0608	0.4433	0.0714	0.2367	0.3405	0.2898	0.8391

* Significant difference; † = variables part of eligibility criteria; *n* = number of visits to schools; *N* = Number of schools; U.P. = usual practice. WASH: Water, sanitation and hygiene.

**Table 4:** Scores for water, sanitation, and hygiene (WASH), menstrual hygiene management (MHM), and WASH+MHM per treatment group in western Kenya, 2012–2013.

Score	Baseline Conditions (Rd 0)					Average Conditions (Rd 1–5)				Change from Baseline to Average Follow-up				Change Across 5 Follow-up Rounds			
	Cup	Pad	U.P.	All Schools	Differ Across Groups	Cup	Pad	U.P.	All Schools	Cup	Pad	U.P.	All Schools	Cup	Pad	U.P.	All Schools
	*n* = 10 *N* = 10	*n* = 10 *N* = 10	*n* = 10 *N* = 10	*n* = 150 *N* = 30		*n* = 50 *N* = 10	*n* = 50 *N* = 10	*n* = 50 *N* = 10	*n* = 150 *N* = 30	*n* = 60 *N* = 10	*n* = 60 *N* = 10	*n* = 60 *N* = 10	*n* = 180 *N* = 30	*n* = 50 *N* = 10	*n* = 50 *N* = 10	*n* = 50 *N* = 10	*n* = 150 *N* = 30
	**Mean, SD (range)**				**CMH *p*-value**	**Mean, SD (range)**				**paired *t*-test, *p*-value**				**Wald *F*-test, *p*-value**			
WASH	1.5, 0.5 (1–2)	1.5, 0.4 (1–2)	1.5, 0.3 (1–2)	1.5, 0.3 (1–2)	0.8656	1.7, 0.8 (0–3)	1.6, 0.8 (0–3)	1.6, 0.8 (0–3)	1.7, 0.8 (0–3)	0.3618	0.4618	0.5597	0.1712	0.3148	0.5429	0.9401	0.0138 *
MHM	1.2, 0.8 (0–2)	1.0, 0.8 (0–2)	1.1, 1.2 (0–3)	1.1, 0.9 (0–3)	0.8347	1.2, 1.0 (0–3)	1.5, 0.9 (0–3)	1.1, 1.1 (0–3)	1.3, 1.0 (0–3)	0.7128	0.1206	0.9128	0.1759	0.5818	0.3589	0.9437	0.2034
WASH + MHM	2.7, 0.9 (1–3.8)	2.5, 0.8 (1–3.5)	2.6, 1.3 (1.5–5)	2.6, 1.0 (1–5)	0.6935	3.0, 1.3 (0–6)	3.1, 1.3 (0–6)	2.7, 1.5 (0–6)	2.9, 1.4 (0–6)	0.2384	0.0793	0.6509	0.0303 *	0.1902	0.8263	0.7369	0.8045

* Significant difference from baseline; *n* = number of visits to schools; *N* = Number of schools; U.P. = usual practice.

**Table 5:** Observed vs. Reported water, sanitation, and hygiene (WASH) variables, average across five follow-up rounds, in western Kenya 2012–2013.

Observed (O) and Reported (R) WASH Indicators	Cup	O vs. R	Pad	O vs. R	U.P.	O vs. R	All	O vs. R
	*n* = 50 *N* = 10	*k*	*n* = 50 *N* = 10	*k*	*n* = 50 *N* = 10	*k*	*n* = 50 *N* =30	*k*
	*n* (%)	*p*-Value	*n* (%)	*p*-Value	*n* (%)	*p*-Value	*n* (%)	*p*-Value
O HW water	42 (84%)	−0.1658, <0.001 *	40 (80%)	−0.2011, <0.001 *	45 (90%)	−0.287, 0.297	127 (84.7%)	−0.1302, <0.001 *
R HW water	45 (90%)		43 (86%)		46 (92%)		134 (89.3%)	
O Soap for HW	13 (26.0%)	0.3544, 0.001 *	14 (28%)	0.320, 0.012 *	15 (30%)	0.3023, 0.016 *	42 (28.0%)	0.3250, <0.001 *
R Soap for HW	31 (62%)		25 (50%)		27 (46%)		83 (55.3%)	
O Washing water in latrines	12 (24.5%)	0.1026, 0.104	14 (28%)	0.1139, 0.156	13 (26%)	0.2336, 0.035 *	39 (26.2%)	0.1447, 0.004 *
R Washing water in latrines	43 (86%)		40 (80%)		30 (60%)		113 (75.3%)	

O = observed; R = reported; All = all schools; *k* = kappa coefficient; *n* = number of visits to schools; *N* = Number of schools; U.P. = usual practice. * Significant difference between observed and reported.

## Supplementary Materials

The following are available online at http://www.mdpi.com/1660-4601/15/8/1682/s1, File S1: Electronic School WASH Survey.
