# Supplementary material for: Do Water, Sanitation and Hygiene Conditions in Primary Schools Consistently Support Schoolgirls’ Menstrual Needs? A Longitudinal Study in Rural Western Kenya
Source: Int J Environ Res Public Health. 2018 Aug 7;15(8):1682. doi: 10.3390/ijerph15081682 (PMC6121484; doi:10.3390/ijerph15081682)
Supplement: Supplementary file 1 [file ijerph-15-01682-s001.pdf]

1424630167

KEMRI/CDC DSS WASH-School survey Form

File number

CI

○ Gem

Visit date __ / __ / ____

Fill for all participating schools

School name: *Schnam*

School ID *SchID*

GIS Location

longitude gis_long

latitude gis_lat

P.O. Box *schbox*

Postal code *pcode*

Town *stown*

Environment: *sch_env* ○ Rural(countryside) ○ Urban(Houses all sides) ○ Periurban(some houses)

Skul oyaw (School open) *sch_open* ☐ Ee (Yes) ☐ Ooyo (No) If No, why ____________ *sch_why*

*If "NO", then the interviewer STOPS here. If "Yes" proceed to Q.1

a) Was water for hand washing observed upon arrival? arrival_hand ☐ Ee (Yes) ☐ Ooyo (No)

b) Was soap seen at handwashing station upon arrival? soap_arrival ☐ Ee (Yes) ☐ Ooyo (No)

1. **Ute mag somo adi mantie e skul?**(How many classrooms are there in the school?) *hwcl_sch*

2. **Adi kuomgi mitiyogo kawuono?**(How many classrooms are in use today?) *hwcl_sch_tdy*

3. **Jopuonj adi mantiere e skul kawuono**?(How many teachers are there at the school) today?(full-time,part-time)?

   Seche duto(FT) *tch_ft*

   Seche moko(PT) *tch_pt*

QUESTION TO TEACHERS:

4. Which teacher helped provide information on WASH today: *wch_teacher*

   ○ Head teacher ○ Full time teacher ○ Part-time teacher ○ Focal point teacher for study

5. **Skul thoro chiwo pii mar luoko lwedo marom nadi(ndik kaka japuonj oduoki pinyka)** hw_often_water
   How often does the school supply water for handwashing?

   ○ Onge(Never) ○ Seche duto(Always) ○ Seche moko(Sometimes):how often (In a month) hw_often_frq

6. **Bende pii mar luoko lwedo nitie kawuono?**(penjoni penj japuonj) (Is there water available for hand-washing today? Is_water

   ○ Ee (Yes) ○ Ooyo (No)

Verified

7. **Skul thoro chiwo sabun mar luoko lwedo marom nadi?(ndik kaka japuonj oduoki pinyka)** *hwsoap_freq*
How often does the school supply soap for handwashing?(write reported frequency below.)

○ Onge(Never) ○ Seche duto(Always) ○ Seche moko(Sometimes) :how often (In a month) ☐☐☐ hw_often_soap

8. **Bende nitiere sabun/pii motimo sabun mar luoko lwedo kawuono?(Penjoni penj japuonj)(** Is there soap/soapy water available for hand washing today(Question to teacher)? Is_soap

○ Ee (Yes) ○ Ooyo (No)

9. How often does the school have cleaning supplies for the latrines?(write reported frequency below.) hw_often_clean

○ Onge(Never) ○ Seche duto(Always) ○ Seche moko(Sometimes) :how often (In a month) ☐☐☐ *hwclean_freq*

10. Are there cleaning supplies available for latrine cleaning today?are_cln_supplies ☐ Ee (Yes) ☐ Ooyo (No)

11. **Bende skul chiwoga gik ler ma nyiri tiyogo ka gidhi e dwe?**(Does the school currently provide sanitary towels for girls? prov_sani
If Yes or sometimes go to Q13

○ Seche moko(Sometimes) ○ Ee (Yes) ○ Ooyo (No)

12. **Ka ooyo,chiw ler gimomiyo ok chiew pamba maler?**(If NO,describe why pads are not given to girls?
pad_notgiven

----

13. **Ka seche moko kata ee,chiw ler ni ka ang'o motimore,kara ang'o,gi I thoro chiwo pamba maler ne nyiri maromo nadi?**(If sometimes or yes,describe under what circumstances,when and how often pads are given to girls? desc_somt ----------------------------------

----

14. **Bende nitie pii ma joma nyiri nyalo luokogo lepgi kapo ni otimo lowo?**(Is there water available for girls to wash if they soil their clothes?iswater_soil
If Yes or sometimes go to Q16

○ Seche moko(Sometimes) ○ Ee (Yes) ○ Ooyo (No)

15. **Ka ooyo,chiw ler gimomiyo ok chiew pii ne nyiri?**(If NO,describe why water isn't available for girls ?
water_notgiven

----

16. **Kaa ee chiw ler(kuom ranyisi:bende pii okan ei choo kata machiegni gi choo mar nyiri**?(If yes or sometimes describe(For example:Is water kept in or near the girls latrine?) if_des

---

OBSERVATION ONLY QUESTIONS

17. **Rang ane ni choche kod kuonde olo pii adi mantiere e skul ?**(Observe how many latrines and urinals with doorway unblocked or unlocked serve the school today?)

Fill table below per every latrine door

User: ***1.Girls*** ***2.Boys*** ***3.Shared*** ***4.Teacher***

Type of latrine*:** ***1.Pit ***2.VIP*** ***3.Ecosan*** ***4.Toilet*** ***5.Urinal***

**User (enter code)**	**Type (enter code)**	**Stable (Y,N)**	**Holes in wall (Y,N)**	**Strong smell (Y,N)**	**Feces or urine(Y,N)**	**Door (Y,N)**	**Door locks inside Y,N**
lat_user	*lat_type*	*lat_stable*	*wall_holes*	*lat_smell*	*lat_urine*	*lat_door*	*lat_lock*

18. ***Observations on Facilities for girls***

a). Are girls latrines in separate bank from boys? *separate_bank* ○ Ee (Yes) ○ Ooyo (No)

b). Is there a privacy wall at girls latrines? *private_latrine* ○ Ee (Yes) ○ Ooyo (No)

c). Is there washing water available today in or very near girls latrines? *washing_water* ○ Ee (Yes) ○ Ooyo (No)

d). Is there a private place where girls can change or wash *girls_change* ○ Ee (Yes) ○ Ooyo (No)

e). If yes to question c or d describe *cd_describe*

---

19. Are there any facilities for hand washing ? *handwash_facility* ○ Ee (Yes) ○ Ooyo (No)
If No go to Q22

20. Observation questions(Fill the table below for containers with water *inside)*

Type: ***1.Bucket 2.Container with tap 3.Leaky tin/tippy tap 4.Other(specify)***

Location: ***1.In girls latrine 2.Near girls latrine 3.Near boys latrine 4.Near latrines,generally 5.Near teachers latrine 6.Near classrooms***

Soap type: ***1.Bar 2.Soapy water 3.Powdered soap 4.Ash 5.Other(specify)***

Cont	Type (enter code)	If other,specify	Location (enter code)	Soap next to container Y,N	If yes soap type:(enter code)	If other,specify
1						
2						
3						
4						
5						
	cont_type	*cont_other*	*cont_loc*	*soap_next*	*soap_type*	*soap_other*

21. Hand washing observation during break-time(11-11:30):

	Boys	Girls	Teachers
**Used facility**	*bfacility*	*gfacility*	*tfacility*
**Washed hands**	*bwash*	*gwash*	*twash*
**Washed hands with soap**	*soapb*	*soapg*	*soapt*

22. If applicable,how are sanitary pads disposed of at the school? sanitary_disposed

- ◯ In pits
- ◯ In buckets(observe)
- ◯ Girls take them back home in bag(observe)
- ◯ Rubbish pit-burned each day
- ◯ Dont know

Others(Mamoko) ______________________
disposed other

23. **Bende nitie gik yweyo/lwoko choo mondo obed maler**(Are there cleaning supplies seen for latrine cleaning? sup_seen

◯ Ee inyalo nee(Yes,seen) ◯ Ee Owachna gi japuonj to ok aneno(Yes,Not seen) ◯ Ooyo (No)

24. **Ka ineno,chan-gi piny ka: (**If seen,list:)seen_list

- -

Other WASH observations *Other_wash*
